# Supplementary material for: Differential guest location by host dynamics enhances propylene/propane separation in a metal-organic framework
Source: Nat Commun. 2020 Nov 30;11:6099. doi: 10.1038/s41467-020-19207-9 (PMC7704659; doi:10.1038/s41467-020-19207-9)
Supplement: Supplementary file 1 — Supplementary Information [file 41467_2020_19207_MOESM1_ESM.pdf]

Differential guest location by host dynamics enhances propylene-propane separation in a metal-organic framework

Dmytro Antypov[1], Aleksander Shkurenko[2], Prashant M. Bhatt[2], Youssef Belmabkhout[2], Karim Adil[2], Amandine Cadiau[2], Mikhail Suyetin[2], Mohamed Eddaoudi[2], Matthew J. Rosseinsky[1], Matthew S. Dyer[1]*

[1] Department of Chemistry, University of Liverpool, Liverpool, UK

[2] King Abdullah University of Science and Technology (KAUST), Physical Sciences and Engineering Division, AMPM Center, Functional Materials Design, Discovery and Development Research Group (FMD3), Thuwal, Saudi Arabia

* e-mail: msd30@liverpool.ac.uk

Supplementary Information

Table of contents

Supplementary Notes 1-3 …… 2

Supplementary Figures 1-18 …… 8

Supplementary Tables 1-7 …… 25

Supplementary References …… 30

Supplementary Note 1: High-pressure gas adsorption studies

High-pressure gas adsorption studies were performed on a magnetic suspension balance marketed by Rubotherm (Germany). Type Adsorption equilibrium measurements of pure gases were performed using a Rubotherm gravimetric-densimetric apparatus G-Hp-Flow (Supplementary Scheme 1), composed mainly of a magnetic suspension balance (MSB) and a network of valves, mass flow meters, and temperature and pressure sensors. The MSB overcomes the disadvantages of other commercially available gravimetric instruments by separating the sensitive microbalance from the sample and the measuring atmosphere, and is able to perform adsorption measurements across a wide pressure range (i.e., from 0 to 20 MPa). The adsorption temperature can be controlled within the range of 77 K to 423 K. In a typical adsorption experiment, the adsorbent is precisely weighed and placed in a basket suspended by a permanent magnet through an electromagnet. Then the cell housing the basket is closed and vacuum or high pressure is applied. The gravimetric method allows the direct measurement of the reduced gas adsorbed amount (Ω). Correction for the buoyancy effect is required to determine the excess and absolute adsorbed amount using equations (1) and (2), where $V_{\text{adsorbent}}$ and V_{ss} and V_{adorbed} phase refer to the volume of the adsorbent, the volume of the suspension system, and the volume of the adsorbed phase, respectively.

$$\Omega = m_{absolute} - \rho_{gas}(V_{adsorbent} + V_{ss} + V_{adsorbed-phase}) \quad (1)$$

$$\Omega = m_{excess} - \rho_{gas}(V_{adsorbent} + V_{ss}) \quad (2)$$

The buoyancy effect resulting from the adsorbed phase may be taken into account via correlation with the pore volume or with the theoretical density of the sample.

These volumes are determined using the helium isotherm method by assuming that helium penetrates in all open pores of the material without being adsorbed. The density of the gas is determined using the Refprop equation of state (EOS) database and checked experimentally using a volume-calibrated titanium cylinder. By weighing this calibrated volume in the gas atmosphere, the local density of the gas is determined. Therefore, simultaneous measurement of adsorption capacity and gas-phase density as a function of pressure and temperature is possible. However, because of the combined low propane uptake and the buoyancy contribution to gravimetric measurement at high pressure, only qualitative kinetic measurements were possible.

The pressure is measured using two Drucks high-pressure transducers ranging from 0.5 to 34 bar and 1 to 200 bar, respectively, and one low pressure transducer ranging from 0 to 1 bar. Prior to each adsorption experiment, about 100 mg of the sample was outgassed at 378 K for 12 hours under a residual pressure of 10^{-6} mbar. The temperature during adsorption measurements is maintained constant using a thermostat-controlled circulating fluid.

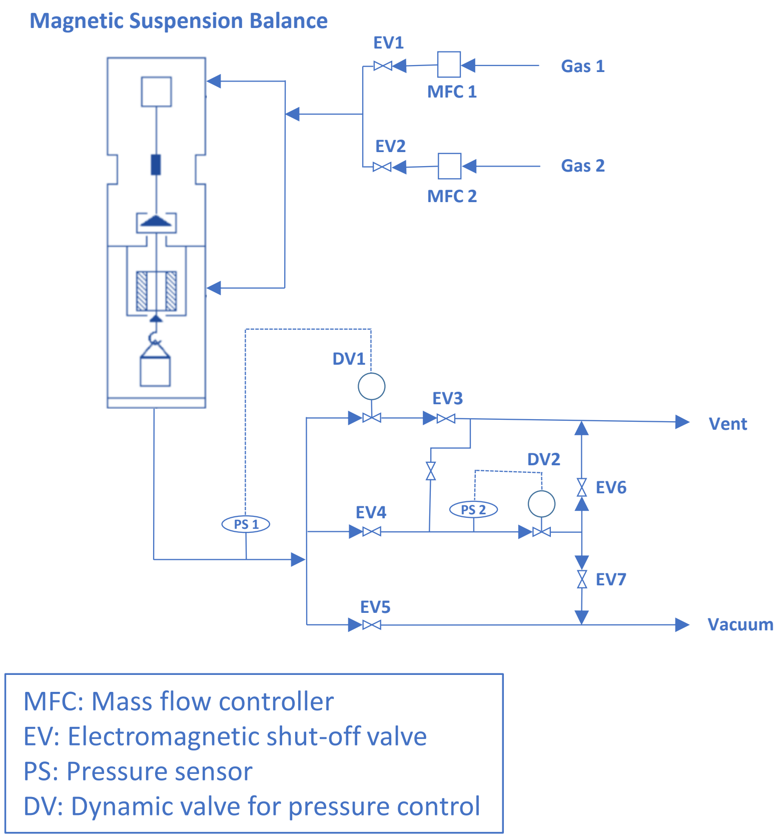

Supplementary Scheme 1. The scheme of the Rubotherm gravimetric-densimetric apparatus.

Supplementary Note 2: Determining the composition of gas mixture adsorbed by NbOFFIVE-1-Ni from C_3H_6/C_3H_8:50/50 gas mixture using gravimetric and GC data.

The GC-coupled mixed gas adsorption was carried out by a custom-made Rubotherm mixed gas gravimetric system coupled with Dani GC. Around 1 g of **NbOFFIVE**-1-Ni MOF was loaded in Rubotherm gravimetric system and heated for 8 hours at 105 °C under dynamic vacuum. **NbOFFIVE**-1-Ni MOF weighed 0.821 g after degassing. A fixed amount of premixed C_3H_6/C_3H_8:50/50 mixture was dosed into the separate mixing chamber at a pressure of 3.6 bar. Then the valve connecting the mixing chamber to the gravimetric system was opened and the adsorption was allowed to equilibrate for 17 hours till the equilibrium was reached. The total volume of the system including mixing chamber and the gravimetric system is 245.33 cm^3. The temperature of the system was controlled and set to 298K. After the equilibrium was reached at 1.3 bar, the non-adsorbed phase was analyzed using GC to determine its composition.

To quantify the composition of gas mixture adsorbed by **NbOFFIVE**-1-Ni after being in contact with a dosed amount of high-grade C_3H_6/C_3H_8:50/50 mixture for 17 hours, we compare the total amount of mixture adsorbed, $Uptake_{gas\ mix}$, obtained from the gravimetric data to the amount of propylene adsorbed, $Ads_{propylene}$, obtained from GC data:

$Uptake_{gas\ mix}$ = The volume in cm^3 the gas mixture adsorbed by the material would occupy at 298 K and 1 bar

$Ads_{propylene}$ = The volume in cm^3 of adsorbed amount of propylene at 298 K and 1 bar

Mass of the sample = 0.821 g

V_{sys} = Effective volume of the system = 245.33 cm^3

P_{eq} = Equilibrium pressure of 1.3 bar at which adsorption was measured relative to 1 bar = 1.3

X = Fractional composition of propylene in a gas mixture before adsorption = 0.5

Y = Fractional composition of propylene in a gas mixture after equilibrium analyzed by GC = 0.452

$Uptake_{gas\ mix}$ = (*Uptake in* $mmol{\cdot}g^{-1}$ * *Mass of the sample*) * 24.5 = (1.53 * 0.821) * 24.5 = 30.73 cm^3,

where *Uptake in* $mmol{\cdot}g^{-1}$ was calculated by using the measured absolute uptake of 64.3 $mg{\cdot}g^{-1}$ (the green point in supplementary Figure 5a) and dividing it by the average molecular weight of the adsorbed gas. Since molecular weight of propylene and propane are similar, 42 and 44 respectively, and propylene is the main adsorbed component 42 was used to calculate *Uptake in* $mmol{\cdot}g^{-1}$ = 64.3 $mg{\cdot}g^{-1}$ / 42 $g{\cdot}mol^{-1}$ = 1.53 $mmol{\cdot}g^{-1}$. The conversion factor of 24.5 $cm^3{\cdot}mmol^{-1}$ is the volume occupied by one mole of ideal gas at 298 K and 1 bar, which is almost the same volume if we use non-ideal equation of state for propylene.

The amount of propylene adsorbed by the material, $Ads_{propylene}$, was calculated as the difference between the amount of propylene initially admitted to the chamber and the amount that still remained in the gas phase after 17 hours of equilibration.

$Ads_{propylene}$ = *(Volume of propylene introduced) – (Volume of residual propylene after adsorption)*

$Ads_{propylene} = X (P_{eq} * V_{sys} + Uptake_{gas\ mix}) - Y (P_{eq} * Vsys)$

$Ads_{propylene}$ = 0.5 (1.3 * 245.33 + 30.73) - 0.452 (1.3 * 245.33)

$Ads_{propylene}$ = 174.83 - 144.15 = 30.68 cm^3

While all measurements used to determine $Uptake_{gas\ mix}$ from gravimetric data are very accurate and the experimental error associated with it is around 0.1%, the main source of error in $Ads_{propylene}$ is due to the low sensitivity of the high-pressure transducer. As it reports pressure values only to one digit after the dot, the actual pressure in the chamber could have been between 1.25 and 1.35 bar. To quantify the error in $Ads_{propylene}$, we recalculated its value by taking the extreme values for P_{eq}:

For P_{eq} = 1.25:

$Ads_{propylene}$ (P_{eq} = *1.25*) = 168.69 - 138.61 = 30.08 cm^3

For P_{eq} = 1.35:

$Ads_{propylene}$ (P_{eq} = 1.35) = 180.96 - 149.70 = 31.26 cm^3

Therefore, for P_{eq} = 1.3 ± 0.05 bar we obtain:

Propylene adsorbed = 30.68 ± 0.6 cm^3

Compared to $Uptake_{gas\ mix}$ = 30.73 cm^3, this allows us to identify

Propylene adsorbed = 99.83 ± 1.95 %

Propane adsorbed = 0.05 ± 0.6 cm^3

Propane adsorbed = 0.16 ± 1.95 %

The uptake of propylene is the same as the uptake of gas mixture within the experimental error.

Supplementary Note 3: Computational Methods

Simulation details. Several Density Functional Theory (DFT) approaches were used in this paper to study the behaviour of two Metal-Organic Framework (MOF) materials, **NbOFFIVE**-1-Ni and **SIFSIX**-3-Ni, with and without propylene or propane guest molecules. We used

- Conjugate gradient energy minimization to identify local energy minimum structures;
- Nudged Elastic Band (NEB) method to identify transition paths between the known energy minima;
- *Ab-initio* Molecular Dynamics (MD) to study the dynamics of the empty host and the host in the presence of a guest molecule close to their equilibrium configuration.

All calculations were performed using the Vienna ab initio Simulation Package (VASP)[1] version 5.4.1 with pseudopotential (POTCAR files) version 54. Most 3D-periodic DFT calculations were performed for a single unit cell containing 4 formula units (Supplementary Scheme 2) with the exception of calculations of determining anion ordering (Supplementary Figure 2) and co-adsorption effects (Supplementary Figure 18) which were performed in a supercell cell containing 8 formula units.

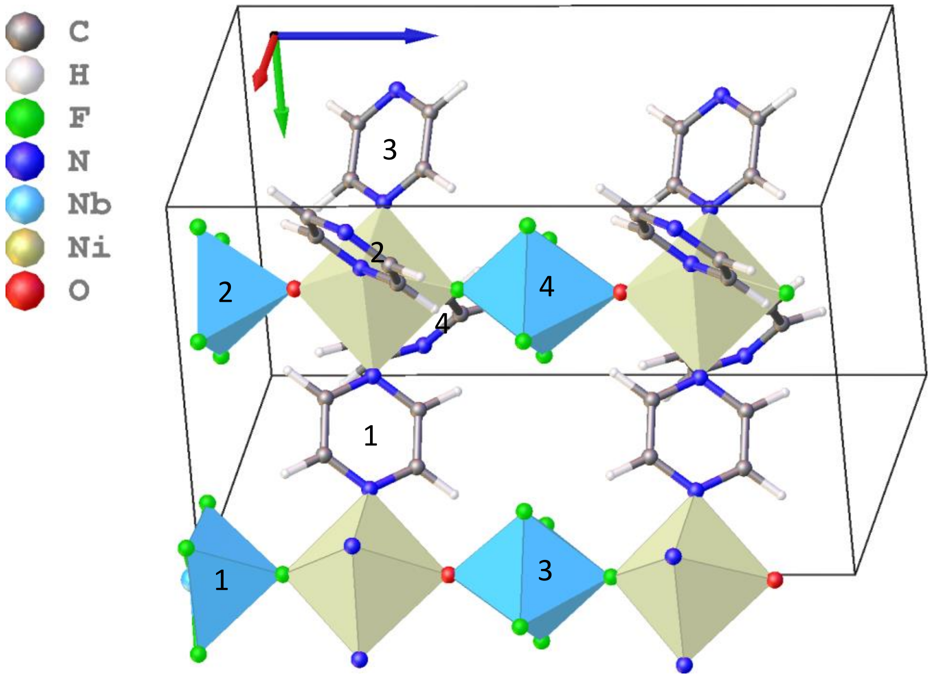

Supplementary Scheme 2. Simulation box set up. The simulation cell containing 4 formula units of $[Ni(NbOF_5)(C_4H_4N_2)_2]$. The blue arrow along the z-axis shows the direction of the two neighbouring 1-D channels running through the cell. The positions of $NbOF_5$ anions at z = 0 (anions 1 and 2) and z = 0.5 (anions 3 and 4) define the positions of two cavities shown in Fig. 1d in the main text. There are two parallel Ni-pyrazine layers in this cell located at fractional coordinates z = 0.25 and z = 0.75. Due to the periodic boundary conditions, the four pyrazine molecules near z=0.25 marked 1 to 4 form a window for each of the two neighbouring 1-D channels running along the z axis.

The optB86B-vdW functional[2] was used in the framework of Projector Augmented Wave method[3] and the Generalised Gradient Approximation.[4] The specific settings used for the VASP calculations were:

- Gaussian smearing of partial orbital occupancies was used with smearing width of 0.1 eV.
- The “normal” precision setting was used with convergence criteria of $1x10^{-6}$ eV for the electronic energy convergence and 10^{-5} eV for the ionic energy convergence.

- Standard potentials with a plane-wave energy cutoff of 520 eV were used in simulations with a flexible cell. For *ab initio* MD calculations in a fixed cell the energy cut off was reduced to its default value of 400 eV.
- The method of conjugate gradients was used to optimise the ion positions, cell volume and cell shape, with a force scaling factor of 0.2. The simulations were typically run for a total of 1000 ionic relaxation steps and periodically restarted to remove the cumulative error in energy arising from varying the cell dimensions.
- The Brillouin zone was sampled using a 2×2×1 Monkhorst-Pack sampling grid.
- Spin-polarization was taken into account for the Ni atoms. While an antiferromagnetic solution was found to be more energetically favorable than the paramagnetic solution, there was no significant difference between the two optimized structures.
- DFT+U method was used with the Hubbard correction of U = 6.45 eV to correct for the self-interaction error introduced by the highly localized d orbitals of Ni atoms.
- In order to obtain fast convergence to the ground state, we set LMAXMIX = 4 so the l-quantum number charge densities of d-electrons are passed through the charge density mixer.

We use the climbing image Nudged Elastic Band calculations (NEB) to capture the energy profile and structural changes associated with guest transport between the known energy minimum configurations. In addition to the two end points, the NEB calculations involved four to twelve intermediate system replicas and used simulation parameters identical to those in our energy minimization calculations. For NEB calculations in a flexible cell the recommended default VASP default settings were used (SPRING = -5, LNEBCELL = .TRUE., IOPT = 3, LCLIMB = .TRUE.). The quasi-Newton (IBRION = 1) algorithm was used to optimize the ionic positions.

We conduct *ab initio* MD to model the system's dynamics to assess host dynamics manifested by the change in the orientation of two structural elements: pyrazine molecules and complex anions, and how the guests affect them. The *ab initio* MD simulations were performed under the constant NVT conditions at 300 K using a time step of 0.5 fs. The temperature was kept constant using the Nose-Hoover thermostat with the period of oscillations set to default 40 times steps (SMASS = 0).

Adsorption energy calculation. The adsorption energy for a guest molecule was calculated as

$$E_{\text{ads}} = E_{\text{host with a guest}} - E_{\text{empty host}} - E_{\text{guest}}$$

where $E_{\text{host with a guest}}$ is the total energy of an optimised cell containing the host and the guest molecule and $E_{\text{empty host}}$ is the energy of the host optimised without the guest present. All atomic positions and the cell geometry were allowed to relax for these two calculations. Note that depending on the orientation of the pyrazine rings there are two possible values for $E_{\text{empty host}}$ – one for identical orientation and one for opposite orientation. The value of $E_{\text{host with a guest}}$ depends on both the orientations of pyrazine rings and the location of the guest in the pore cavity. The adsorption energies quoted in the main text correspond to the lowest energy configurations observed in calculations, with full detail of all metastable states given in Supplementary Table 6. The energy of a free guest molecule, E_{guest}, was calculated for a single guest molecule. Since a plane wave basis sets method assumes the use of periodic boundary conditions, a relatively large cubic box containing a single molecule was used to represent the gas phase. We found that the box size of 15 Å was large enough to ensure that the interactions with the periodic images were below 10^{-6} eV.

A similar approach was used to assess the effect of thermal motion on adsorption energy by running three separate ab initio MD simulations and calculating time-averaged energy values for a host with a guest, empty host and a guest in a gas phase:

$$E_{\text{ads at 300K}} = \langle E_{\text{host with a guest}} \rangle - \langle E_{\text{empty host}} \rangle - \langle E_{\text{guest}} \rangle$$

Here time-averaging for all three reference systems is performed over a simulation time of at least 10 ps.

Attractive host-guest interactions produce conformational changes in both the guest and the host that we quantify as strain energies to explain the role of these interactions for each adsorption site. The guest strain, S_{guest}, is calculated as the difference between the guest energy at the adsorption site $E_{\text{guest ads}}$ and the energy of a free guest:

$$S_{\text{guest}} = E_{\text{guest ads}} - E_{\text{guest}}$$

Similarly, the framework strain, S_{host}, is calculated as the difference between the host energy at the adsorption site $E_{\text{host ads}}$ and the empty host:

$$S_{\text{host}} = E_{\text{host ads}} - E_{\text{empty host}}$$

The strain values are always positive and represents the conformational changes to either the guest or the host due to their interaction.

Both $E_{\text{guest ads}}$ and $E_{\text{host ads}}$ are calculated without structural relaxation for the exact atomic positions identified for the adsorption site. We also define attractive host-guest interactions for a given adsorption site as

$$E_{\text{host-guest attraction}} = E_{\text{host with a guest}} - E_{\text{host ads}} - E_{\text{guest ads}}$$

which is different from E_{ads} as it does not include stress energies:

$$E_{\text{host-guest attraction}} = E_{\text{ads}} - S_{\text{host}} - S_{\text{guest}}$$

Supplementary Table 6 shows the values of E_{ads}, S_{host}, S_{guest}, and $E_{\text{host-guest attraction}}$ for all adsorption sites discussed in the main text. Supplementary Table 7 compares the adsorption energies E_{ads} of global minima to average adsorption energies $E_{\text{ads at 300K}}$ calculated in *ab initio* MD simulations.

Supplementary Figures

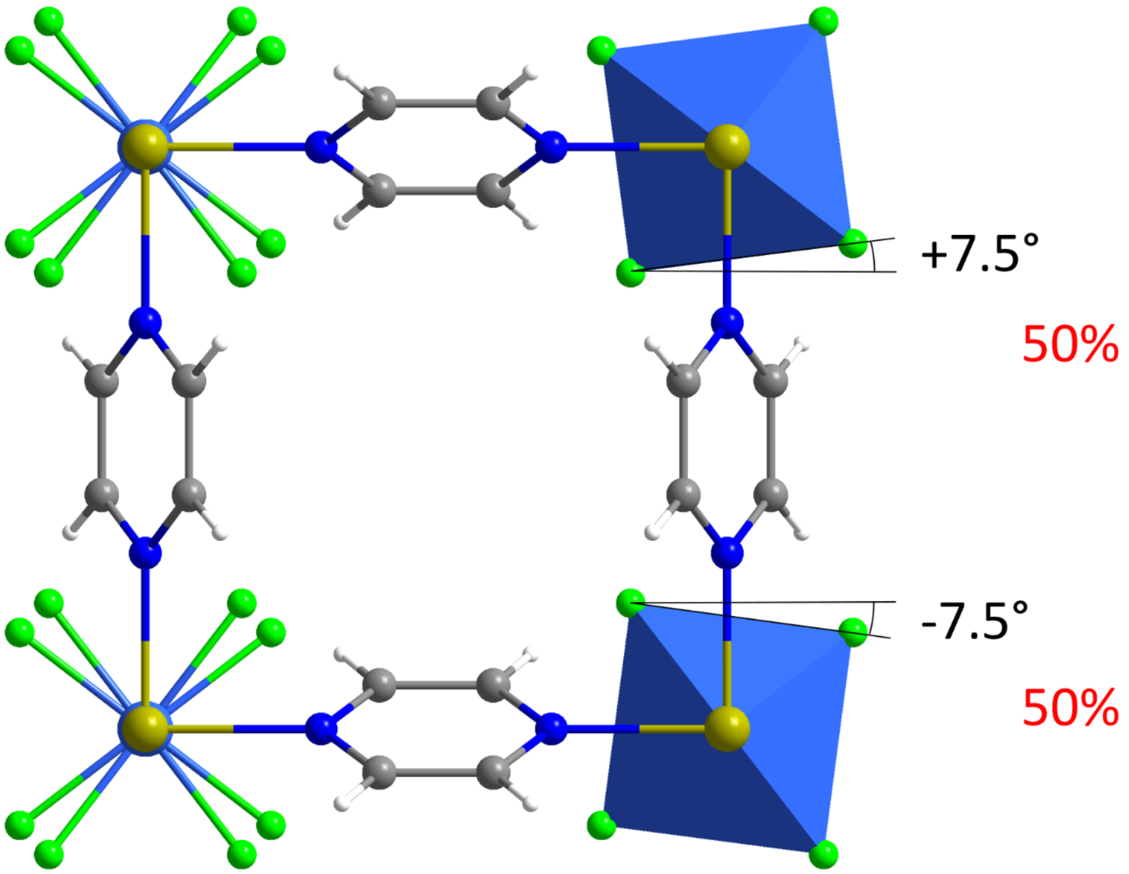

Supplementary Figure 1. Anion disorder in desolvated NbOFFIVE-1. Two possible $(NbOF_5)^{2-}$ orientations in the activated **NbOFFIVE**-1 crystal structure (left part of the structure) and two different selected orientations with the angle of rotation and occupancy indicated next to each anion (right part of the structure).

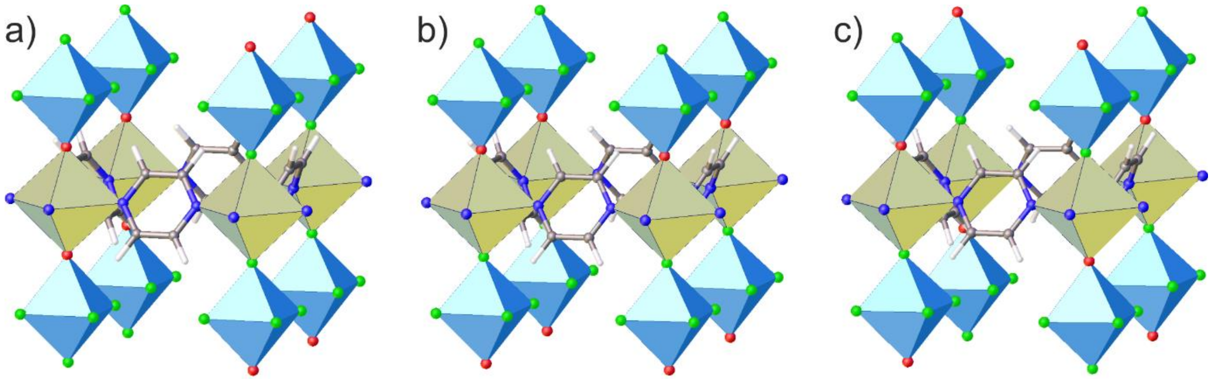

**NbOFFIVE**-1-Ni structure model	***V*, Å³**	***E*, eV**	**Δ*E*, kJ/mol**
O/F ordering a) with opposite pyrazines	3036.5	-1210.030	2.4
O/F ordering b) with opposite pyrazines	3014.9	-1210.065	2.0
O/F ordering c) with opposite pyrazines	3017.6	-1210.035	2.3
O/F ordering a) with identical pyrazines	3030.4	-1210.084	1.7
O/F ordering b) with identical pyrazines	3020.4	-1210.086	1.7
O/F ordering c) with identical pyrazines	3017.6	-1210.229	set to 0

Supplementary Figure 2. **Identification of the lowest energy structure for NbOFFIVE-1-Ni**. A series of DFT optimisation calculations using a **NbOFFIVE**-1-Ni supercell containing 8 formula units were performed for different orientations of $(NbOF_5)^{2-}$ anions (shown as blue octahedra) defined by the positions of axial O (red) and F (green) atoms. Only one of the two Ni-pyrazine layers occupying the simulation cell is shown for clarity. Two orientations of pyrazine molecule in the second layer (not shown) were considered – one identical to that of pyrazines in the first layer and one in which all pyrazines had the opposite tilt. The cell volumes, total energies and the relative energy per formula unit are shown for two types of pyrazine ordering and for three distinct patterns of O/F axial atoms. In a) each Ni atom, shown as a pale yellow octahedron, is coordinated to either two oxygen atoms or two fluorine atoms. In b) and c) each Ni atom is coordinated to one oxygen and one fluorine atom – in b) all $(NbOF_5)^{2-}$ anions have identical orientation, while in c) their orientations alternate. Both the pyrazine ordering and the location of axial F/O atoms on $(NbOF_5)^{2-}$ anions is shown to have a small effect on the thermodynamic stability of the structure as indicated by small energy differences between the optimised structures (the accuracy of the energy convergence is around 0.1 kJ/mol per formula unit). For our further calculations we used the lowest energy structure c) with strictly alternating positions of axial O/F atoms and identical orientations of equivalent pyrazines molecules in adjacent Ni-pyrazine layers.

All anions in the minimum energy configuration are rotated by $\varphi = 10.2°$ due to the unequal interactions with the adjacent pyrazine layers. Due to the symmetry of the lowest energy structure, a smaller cell (Supplementary Scheme 2) containing 4 formula units was used in the rest of this paper.

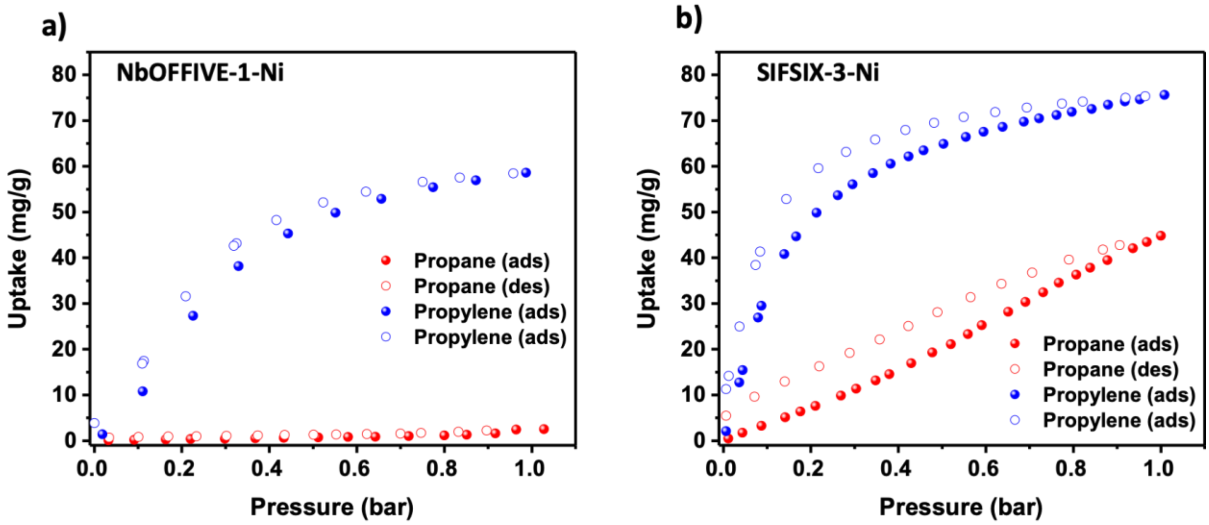

Supplementary Figure 3. Low pressure adsorption data. Low pressure propane and propylene adsorption isotherms (T = 298 K) in a) **NbOFIVE**-1-Ni and b) **SIFXSIX**-3-Ni.

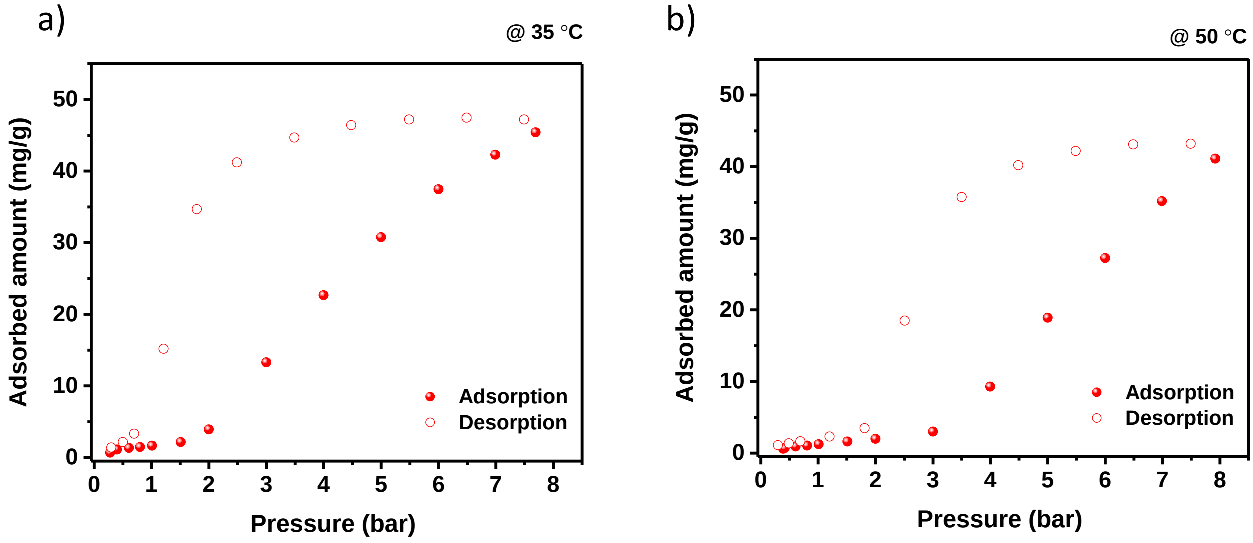

Supplementary Figure 4. High pressure adsorption data for propane in NbOFFIVE-1-Ni. Propane adsorption on **NbOFFIVE**-1-Ni at a) 35°C and b) 50°C.

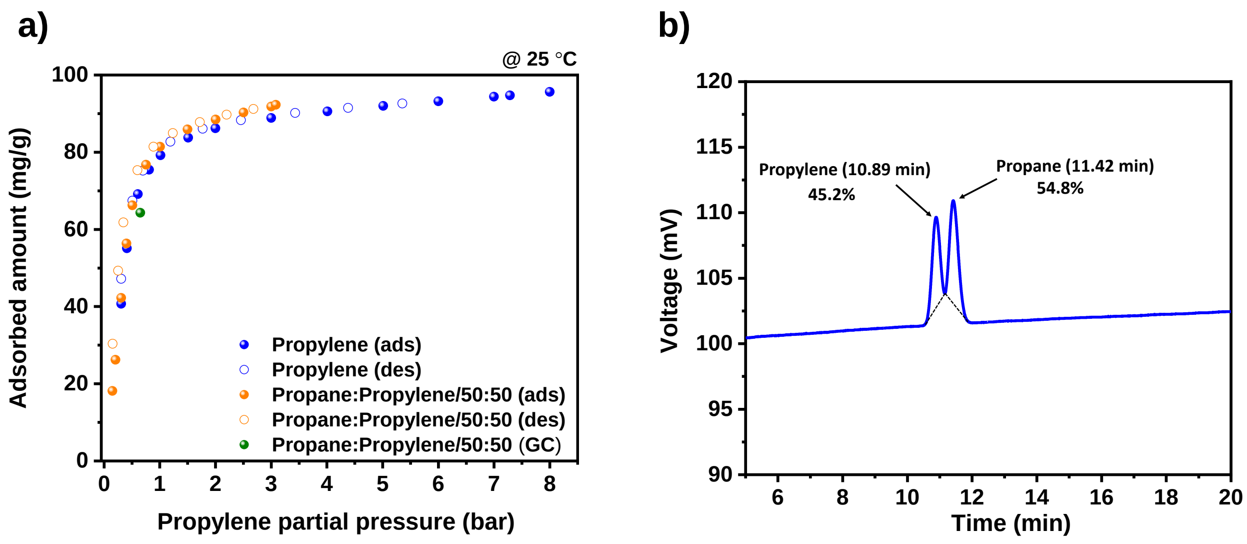

Supplementary Figure 5. High-pressure adsorption data and GC analysis of non-adsorbed phase in NbOFFIVE-1-Ni. (a) High-pressure adsorption isotherms at 25 °C for pure propylene (blue) and a C_3H_6/C_3H_8:50/50 mixture (orange) compared with a single GC-coupled mixed gas adsorption measurement for C_3H_6/C_3H_8:50/50 at 1.3 bar total pressure. The difference between the blue and the orange curve at any given partial pressure of propylene is indicative of the small amount of propane co-adsorbed from the mixture (b) GC analysis of the non-adsorbed phase after adsorption of C_3H_6/C_3H_8:50/50 on **NbOFFIVE**-1-Ni, showing that the composition of gas mixture has changed from 50/50 to 45.2/54.8 due to selective adsorption of propylene by the material.

a)

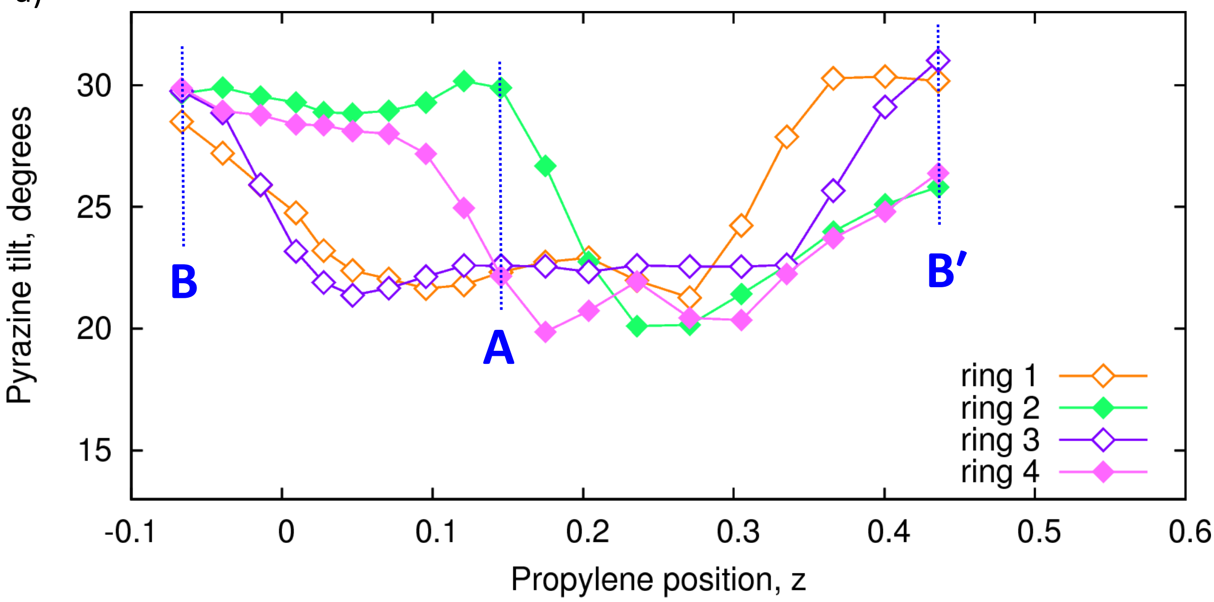

b)

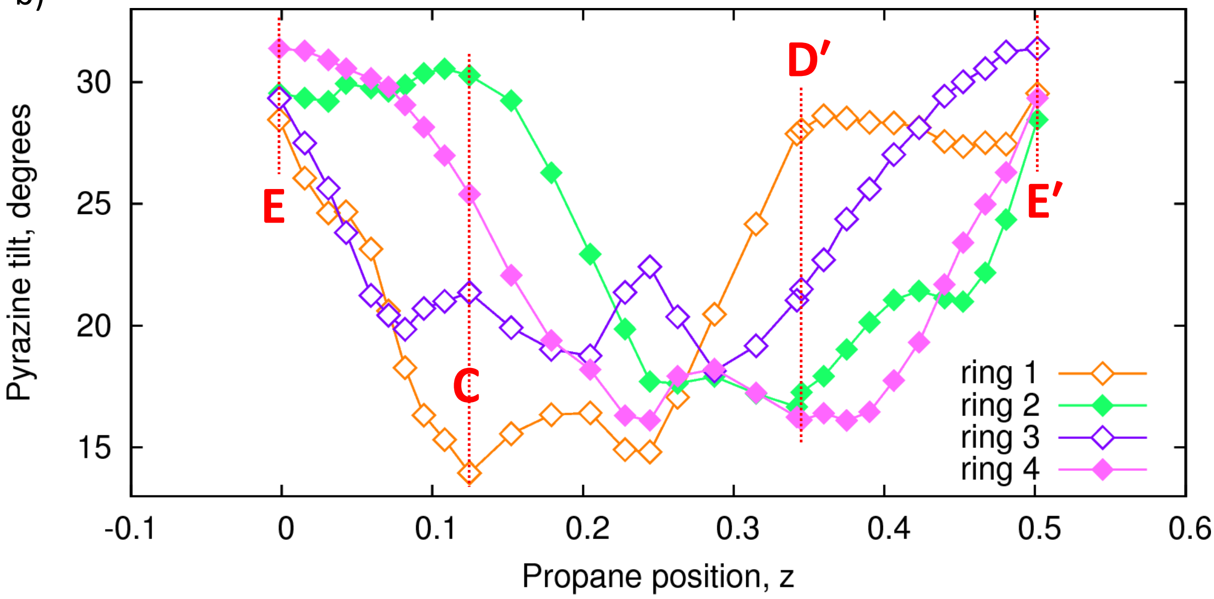

Supplementary Figure 6. Calculated pyrazine tilts. The tilts of four pyrazine rings comprising the window observed in calculated diffusion pathways (Supplementary Scheme 2) are shown as a function of the guest position in **NbOFFIVE**-1-Ni structure for a) propylene and b) propane. The position of the guest z is defined by the fractional coordinate of its central carbon. The four pyrazine rings are located at z = 0.25, whereas z = 0 and z = 0.5 correspond to the centres of the two adjacent cavities. The rings are numbered such that rings 1 and 3 are opposite each other and so are rings 2 and 4 (Supplementary Scheme 2). The tilt of pyrazine rings in the optimised empty structure is θ= 29.2°. As the methyl group on either guest approaches the window, the tilts of pyrazine rings 1 and 3 decrease ($0 < z < 0.05$) followed by rotation of ring 4 that leads to metastable configuration A and C. To allow guest transport through the window at z = 0.25, ring 2 also needs to rotate – the ring rotation is more pronounced in the case of propane, compared to propylene.

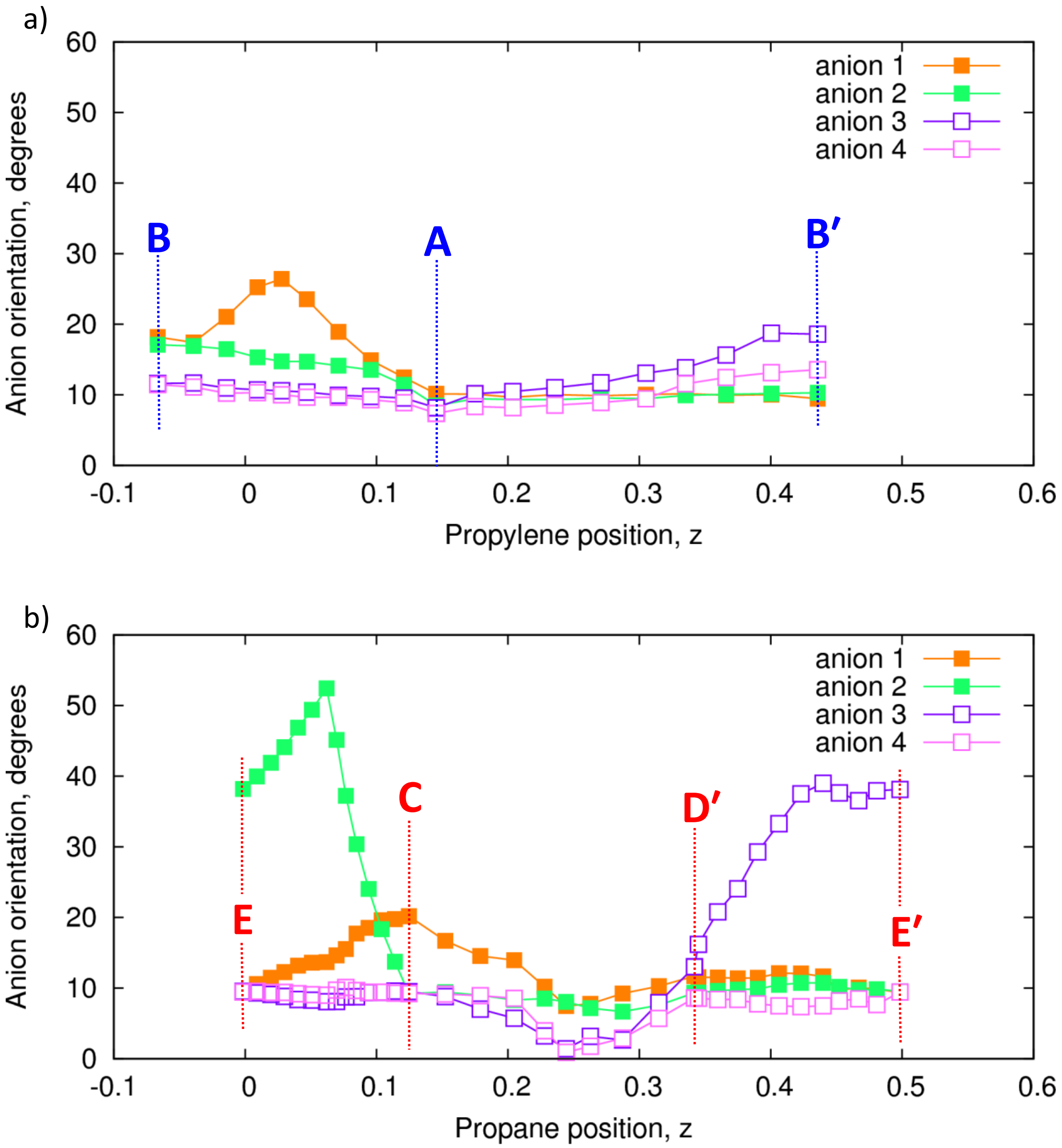

Supplementary Figure 7. **Calculated anion rotations tilts.** The orientation of all four anions present in the cell along the calculated diffusion pathways (Fig. 2 of the main text) are shown as a function of the guest position in **NbOFFIVE**-1-Ni structure for a) propylene and b) propane. The position of the guest *z* is defined by the fractional coordinate of its central carbon. The anions 1 and 2 are located at *z* = 0 in cavity 1, whereas anions 3 and 4 are located at *z* = 0.5 in cavity 2. Anions 1 and 2 and their two periodic images form cavity 1 (Fig. 2 of the main text). The orientation of anions in optimised empty structure is φ = 9.1°. The relatively small deviations of anion orientations from this value in configurations A, B, C and D′ are linked to the changes in tilts of pyrazine molecules they form a hydrogen bond with and to the excluded volume interactions with the guests. There is a large change in the orientation of exactly one anion when propane approaches the global minimum configuration in the centre of cavity 1 (*z* = 0) or cavity 2 (*z* = 0.5).

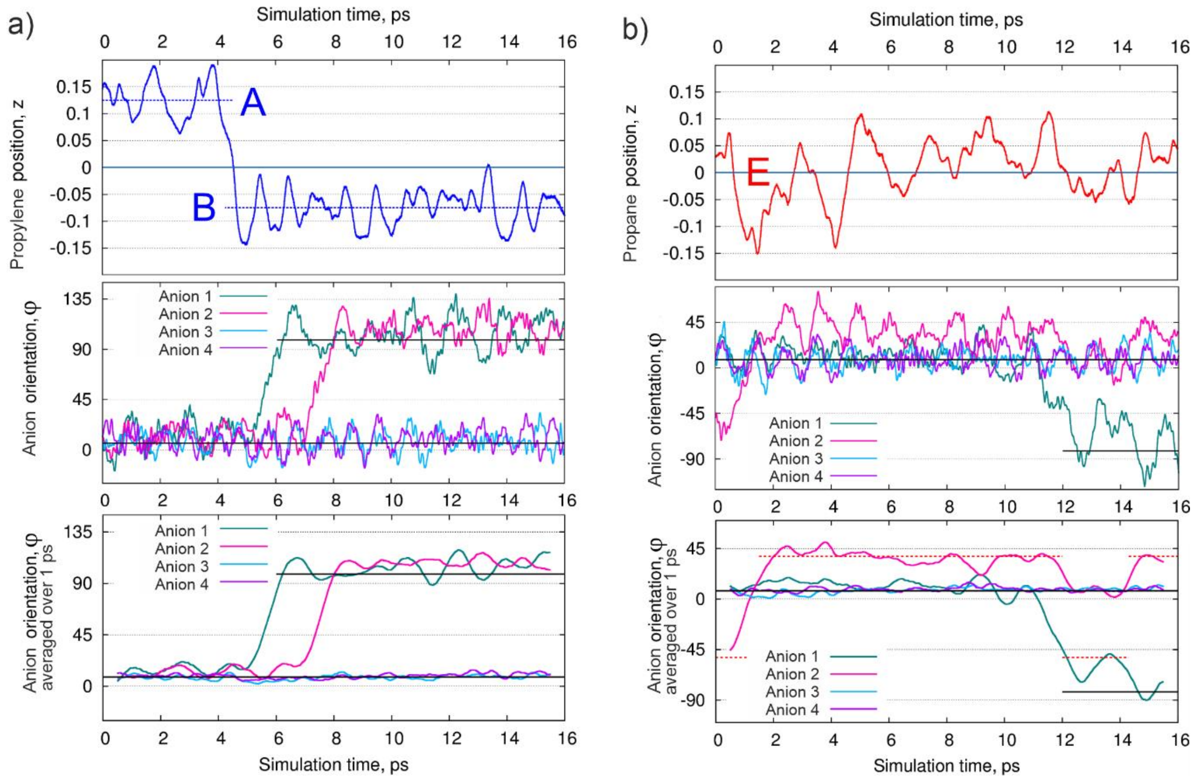

Supplementary Figure 8. Guest position and anion orientation from a 16 ps MD trajectories. Guest position *z* (top) and the orientation φ of four $(NbOF_5)^{2-}$ anions (raw data in the middle panel and the same data averaged over 1 ps at the bottom) as a function of simulation time in *ab initio* MD calculations at 300 K for a) propylene and b) propane in **NbOFFIVE**-1-Ni. The horizontal blue line at *z* = 0 indicates the location of the centre of the cavity occupied by the guest. The propylene molecule resides at one of the cavity ends – it leaves the metastable adsorption site (configuration A) at *z* = 0.13 after about 4 ps and spends the remaining 12 ps of the simulation at the lowest energy adsorption site at *z* =-0.07 (configuration B that is also observed experimentally). The propane molecule oscillates around the centre of the cavity, *z* = 0, (configuration E that is also observed experimentally). The orientation of the octahedral $(NbOF_5)^{2-}$ anions, φ, is defined with respect to the square grid of the Ni-pyrazine layers (see Fig. S4) with $\varphi = 7.3°$ corresponding to the anion orientation in the empty host at 300 K shown as solid black lines in the bottom panels. The spontaneous rotation of the $(NbOF_5)^{2-}$ anions by 90° to an energetically equivalent configuration can be seen for both propylene (at 5 ps for anion 1 and at 7 ps for anion 2) and propane (at 1 ps for anion 2 and at 11 ps for anion 1). The equilibrium orientations of anions 1 and 2 that are in close proximity to the guest change as the result of these interactions compared to anions 3 and 4 in the neighbouring empty cavity. In the case of propylene both anions are affected by a small amount of 7°, whereas in the case of propylene the equilibrium orientation one anion is affected at a time by a large amount of over 30°. The red dashed line in (b) shows the equilibrium orientation $\varphi = 38°$ of the anion currently neighbouring the propane guest and is different from equilibrium orientation $\varphi = 7.3°$ (black line) due to the attractive host-guest interactions.

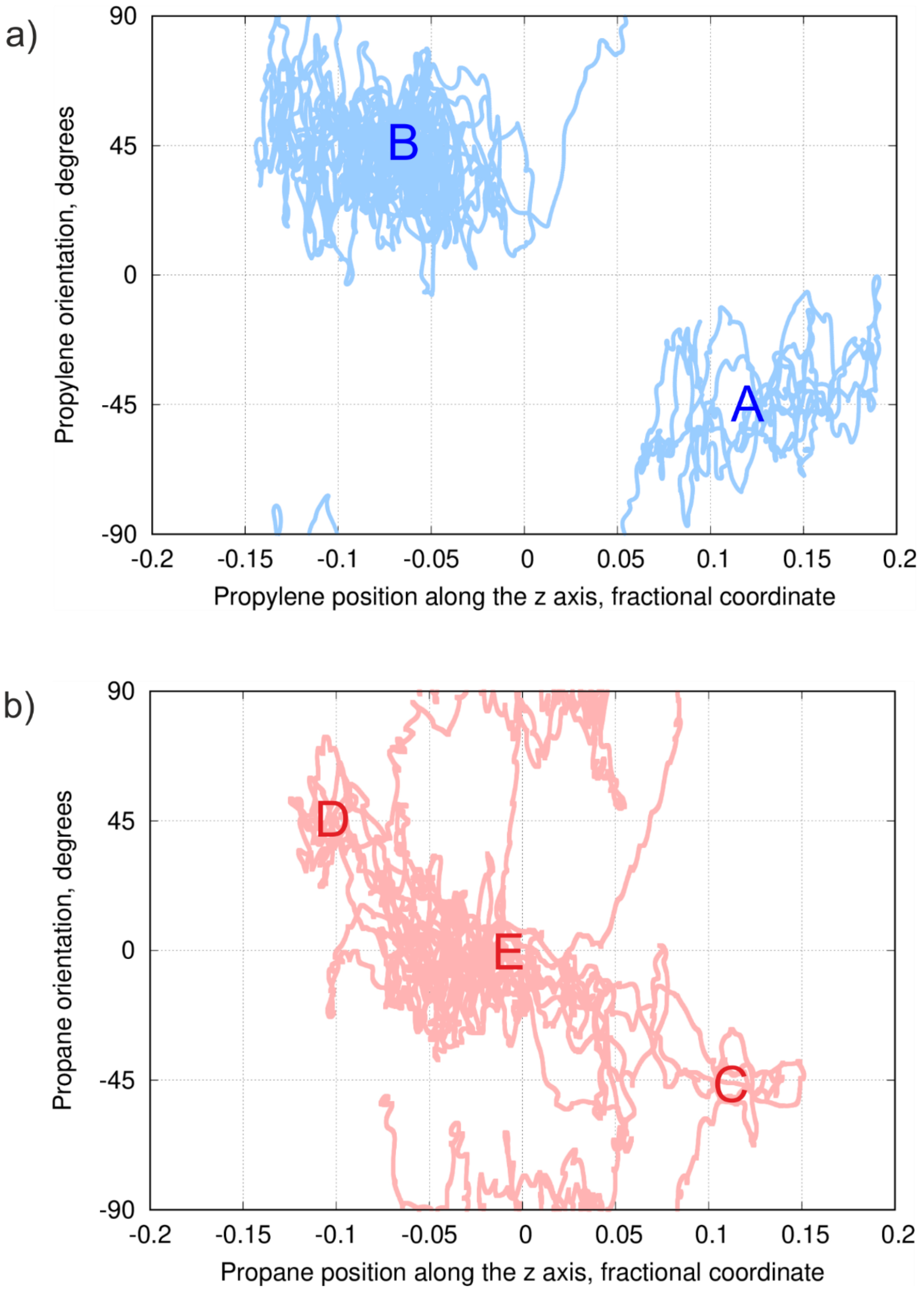

Supplementary Figure 9. Guest position and orientation in NbOFFIVE-1-Ni. The orientation of a) propylene molecule and b) propane molecule as a function of their position in the pore cavity from the 16 ps MD simulation trajectories shown in Supplementary Figure 8. The guest orientation is defined by the normal vector to the plane defined by the three carbons such that -45° and +45° correspond to this vector being parallel to the square grid of the Ni-pyrazine layers (Figure 2 of the main text), while 0° and ±90° correspond to the orientation of the guest along the diagonal of the grid (Figure 3c of the main text).

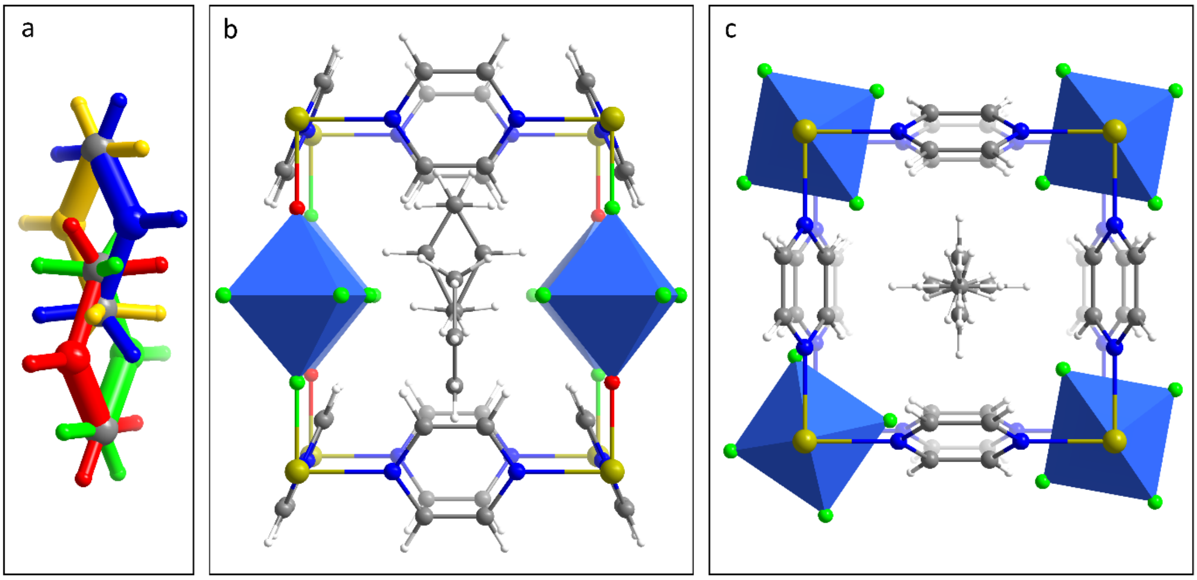

Supplementary Figure 10. Guest molecule disorder in NbOFFIVE-1-Ni·0.85(propylene) crystal structure. a, Four different orientations of a propylene molecule in the pore cavity of **NbOFFIVE**-1-Ni. **b**, Side view and **c**, top view on the cavity with the disordered propylene molecule.

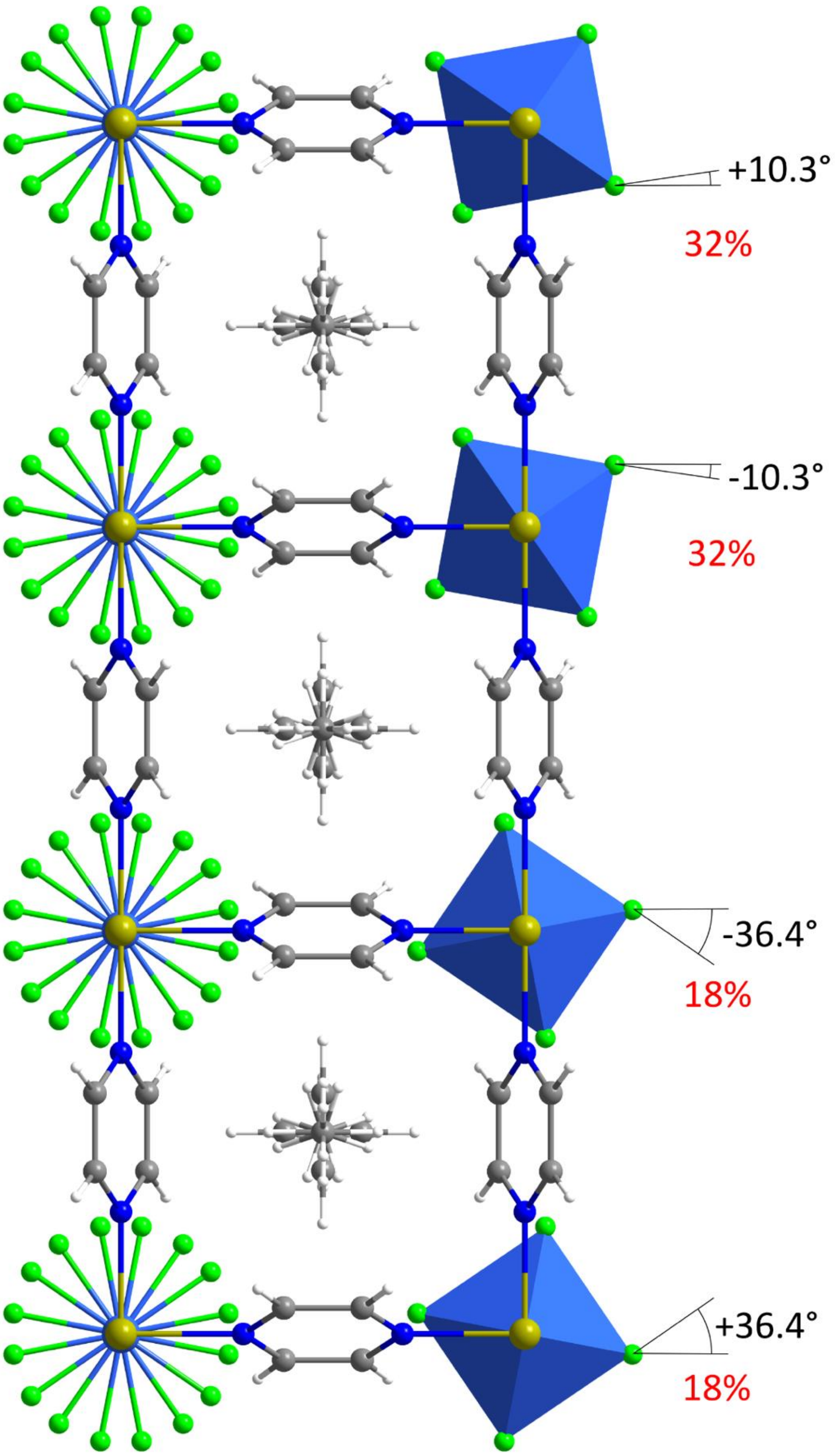

Figure 11. Anion disorder in NbOFFIVE-1-Ni·0.85(propylene) crystal structure. All possible $(NbOF_5)^{2-}$ orientations in **NbOFFIVE**-1-Ni·0.85(propylene) crystal structure (left part of the structure) and selected orientations with the angle of rotation and occupancy (right part of the structure).

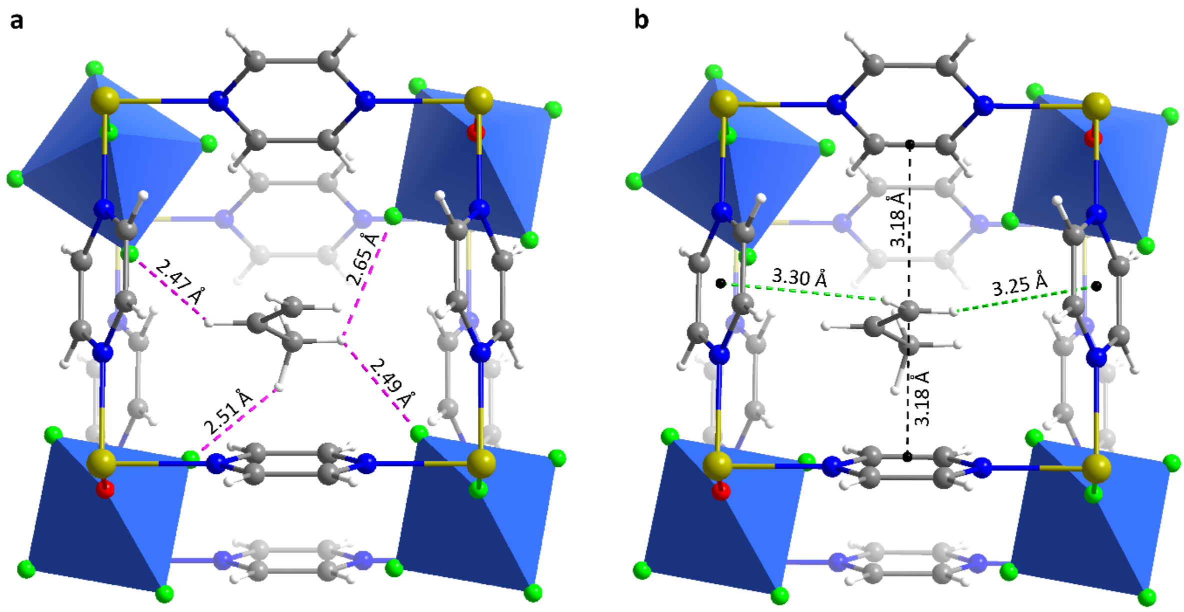

Figure 12. Guest-host interactions in the NbOFFIVE-1-Ni·0.81(propane) crystal structure. Intermolecular propylene-MOF interactions in **NbOFFIVE**-1-Ni·0.85(propylene) crystal structure: **a**, C–H···F contacts in pink; **b**, C–H···π in green and π-π interactions in black.

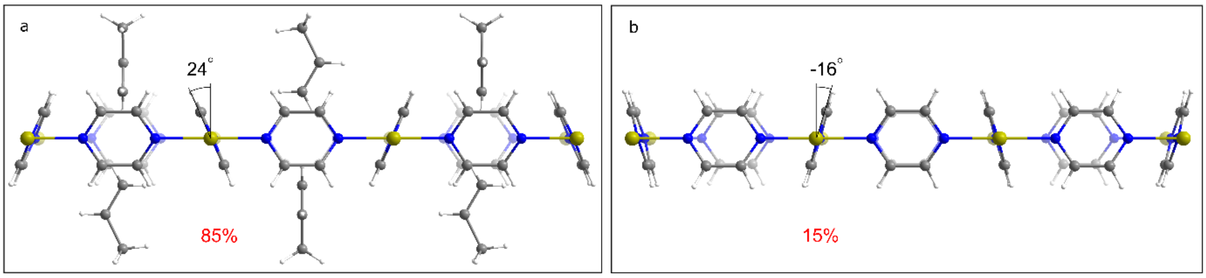

Supplementary Figure 13. Pyrazine disorder in NbOFFIVE-1-Ni·0.85(propylene) crystal structure. Two kinds of Ni(pyrazine)$_2$ layers in the **NbOFFIVE**-1-Ni·0.85(propylene) crystal structure: **a**, the layers which can include propylene molecules (85%); **b**, the layers which do not include propylene molecules (15%).

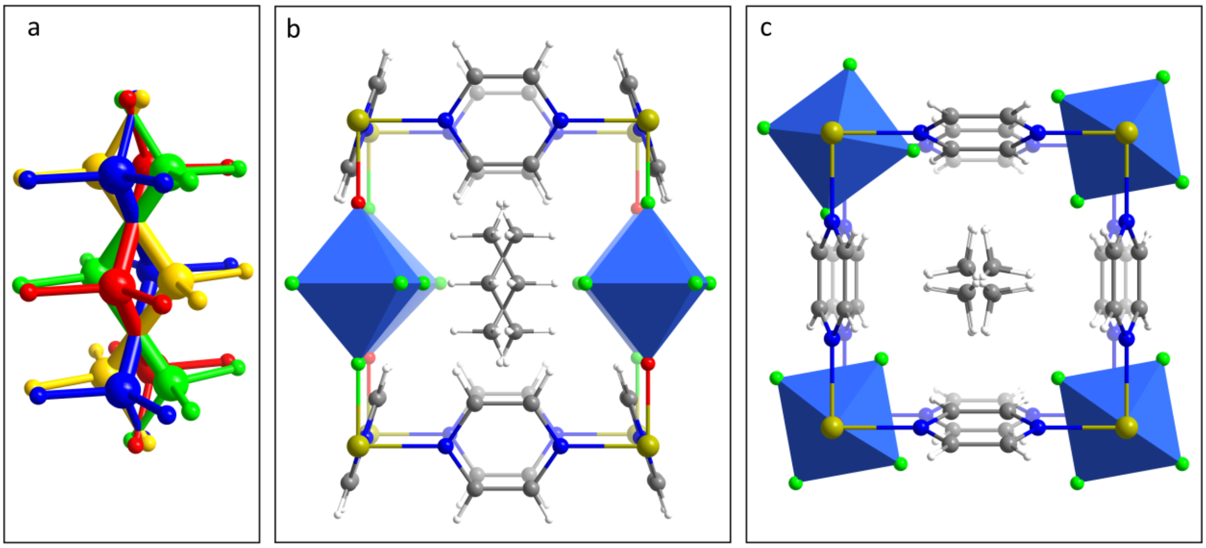

Figure 14. Guest molecule disorder in NbOFFIVE-1-Ni·0.81(propane) crystal structure. a, Four different orientations of a propane molecule in the pore cavity of **NbOFFIVE**-1-Ni. **b**, Side view and **c**, top view on the cavity with the disordered propane molecule.

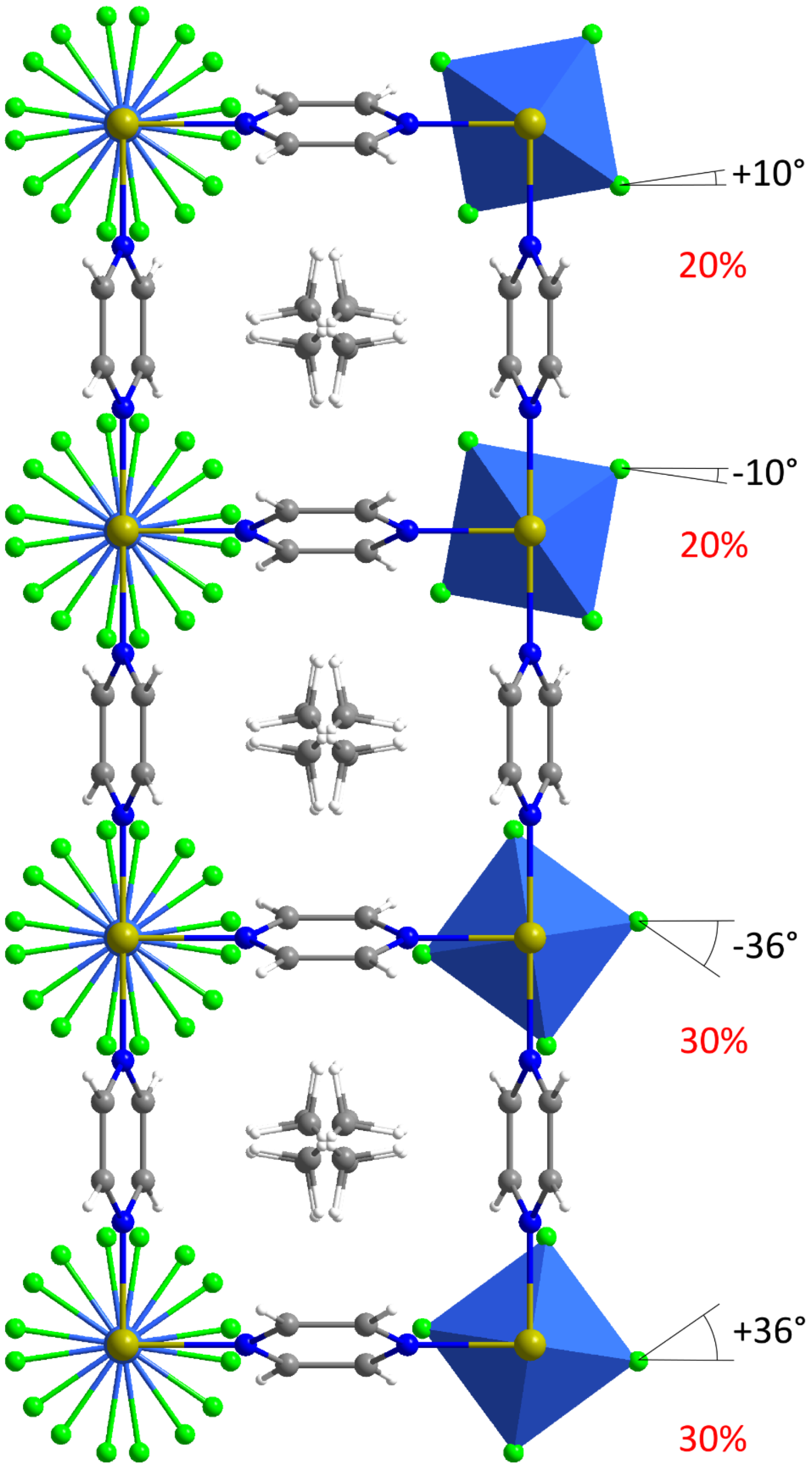

Figure 15. Anion disorder in NbOFFIVE-1-Ni·0.81(propane) crystal structure. All possible orientations of $(NbOF_5)^{2-}$ anions in the **NbOFFIVE**-1-Ni·0.81(propane) crystal structure (left) and selected orientations with the angle of rotation and occupancy (right).

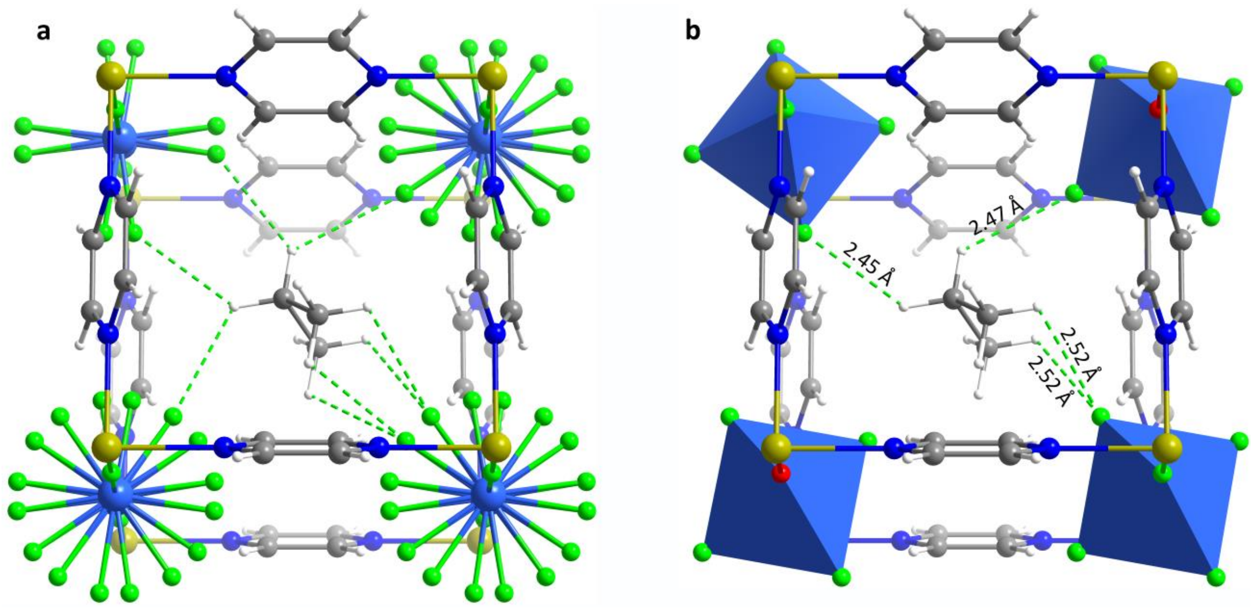

Figure 16. C–H···F contacts in the NbOFFIVE-1-Ni·0.81(propane) crystal structure. **a**) All possible anion orientations and **b**) selected anion orientations that show rotation of a single anion are shown.

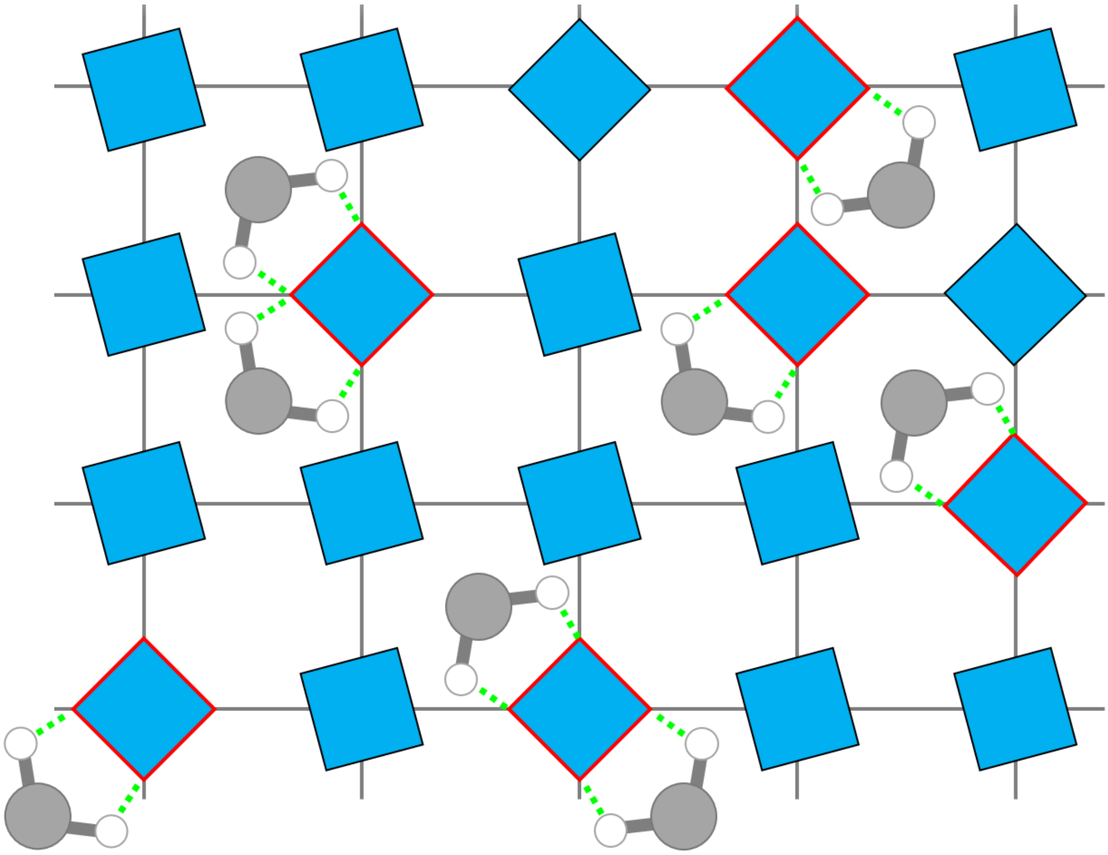

Supplementary Figure 17. The random distribution of induced anion rotations. The diagram illustrating a cross section through the layer of **NbOFFIVE**-1-Ni in which p = 50% of the cavities are occupied by propane molecules (only the central carbon and its two hydrogen atom are shown for clarity). Each propane molecule causes rotation of exactly one of the four neighbouring anions (blue) forming cavity walls: the rotated anions are highlighted in red. Since each anion is shared between four neighbouring cavities, some of the anions will interact with more than one propane molecule. This means that the fraction of rotated anions will be less than p.

Since our *ab initio* MD simulations indicate that a propane molecule diffuses between the four anions forming the cavity on a picosecond time scale, it is reasonable to assume that, over the time scale of the SCXRD experiment, each of the four anions will be rotated a quarter of the time due to the presence of the propane molecule in the cavity. Therefore, the probability that a given anion, shared by four cavities each occupied with probability p = 0.808 by a propane molecule, retains its unperturbed equilibrium orientation is $(1 - p/4)^4 = 0.406$. The probability of anion being rotated is equal to 1 - 0.406 = 0.594 that compares well to the experimentally observed value of 60%.

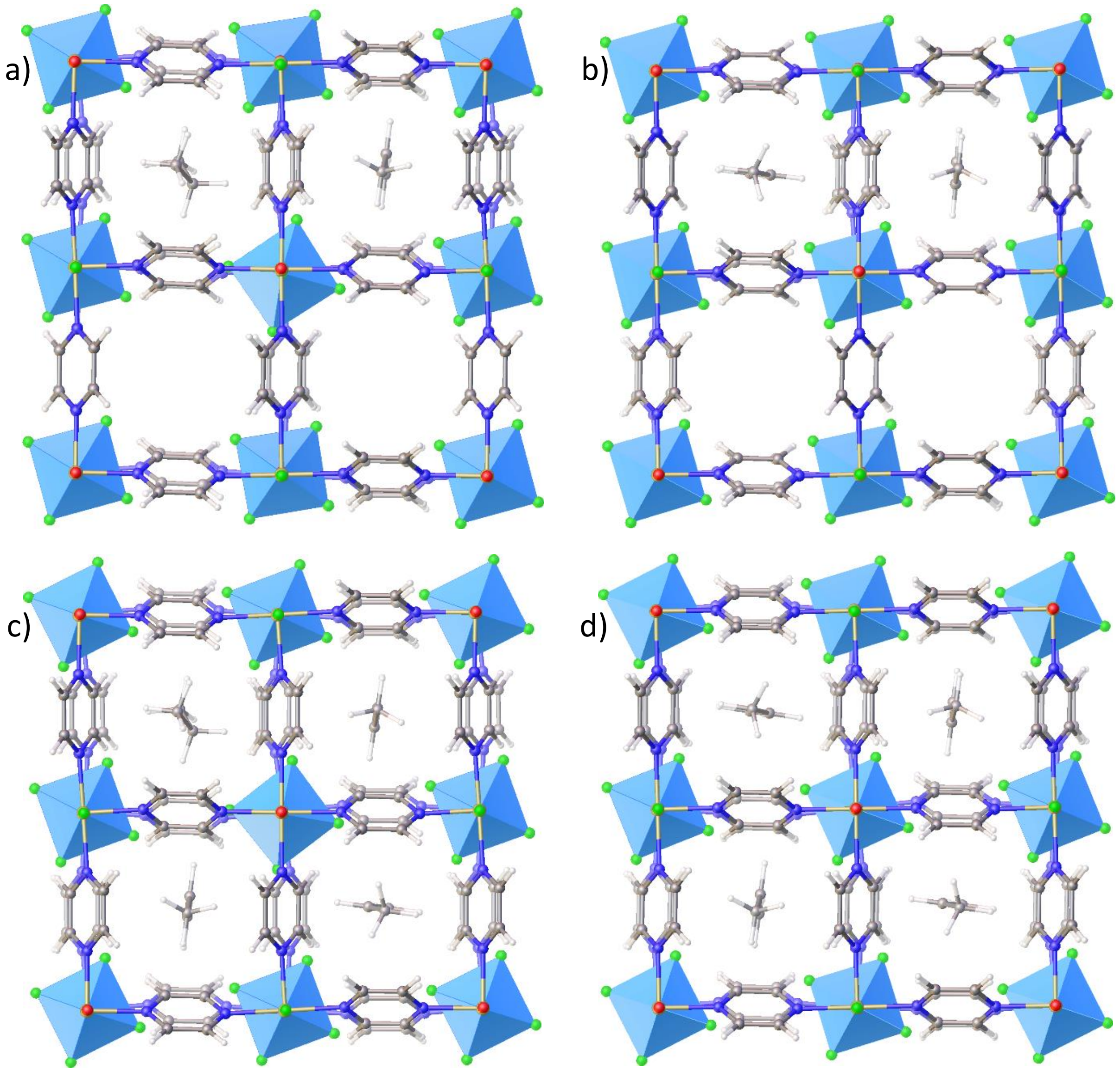

Supplementary Figure 18. Computational assessment of propane co-adsorption. Lowest energy configurations were identified for 2x1x1 supercells containing several guest molecules in the neighbouring cavities: a) one propane and one propylene molecule b) two propylene molecules c) one propane and three propylene molecule d) four propylene molecules. The anion in the centre of a) and c) has a different orientation compared to other anions in the system due to the interaction with the propane molecule that is located near it. In the absence of propane, all anions in b) and d) have the same orientation. By comparing total binding energies, it is possible to tell that adsorption of a propylene molecule instead of propane is energetically favourable in b) compared to a) by ΔE=13.4 kJ/mol and in d) compared to c) by ΔE=8.0 kJ/mol. If we approximate the propylene/propane ratio in the material by that of a two-state statistical model, $e^{\Delta E/kT}/1$, and use these two calculated differences in binding energies, we obtain an estimate for the equilibrium compositions of propylene and propane in the adsorbed phase at T=298K as respectively 99.6/0.4 for the loading of 0.5 and 96.2/3.8 for full loading. While this Boltzmann population analysis is approximate, our simulations clearly indicate that (1) the propane is located opposite the anion and the anion rotation still takes place even when all neighbouring cavities are occupied by propylene, (2) regardless of the pore loading, there is a thermodynamic preference to adsorb propylene instead of propane and (3) this preference reduces as the loading increases which is expected to increase the trace of propane in the

adsorbed phase as the uptake increases with pressure at a given temperature. These results are in qualitative and quantitative agreement with the available experimental data.

Supplementary Tables

Supplementary Table 1. Crystal data and structure refinement conditions for NiNb_empty

Empirical formula	$C_8H_8F_5N_4NbNiO$
Formula weight	422.80
Crystal system, space group	Tetragonal, *P*4/*nbm*
Unit cell dimensions	a = 9.9291(3) Å, c = 7.8311(4) Å
Volume	772.04(6) Å^3
Z, calculated density	2, 1.819 Mg m^{-3}
F(000)	412
Temperature (K)	296(2)
Radiation type, λ	Cu $K\alpha$, 1.54178 Å
Absorption coefficient	8.10 mm^{-1}
Absorption correction	Multi-scan
Max and min transmission	0.149 and 0.030
Crystal size	0.01 × 0.01 × 0.02 mm
Shape, colour	Prism, colourless
θ range for data collection	6.3–66.5°
Limiting indices	$-9 \leq h \leq 10$, $-11 \leq k \leq 11$, $-3 \leq l \leq 9$
Reflection collected / unique / observed with $I > 2\sigma(I)$	2094 / 375 (R_{int} = 0.035) / 297
Completeness to θ_{max} = 66.5°	99.2 %
Refinement method	Full-matrix least-squares on F^2
Data / restraints / parameters	375 / 6 / 31
Final R indices [$I > 2\sigma(I)$]	R_1 = 0.039, wR_2 = 0.106
Final R indices (all data)	R_1 = 0.046, wR_2 = 0.113
Weighting scheme	$[\sigma^2(F_o^2) + (0.0743P)^2 + 0.2648P]^{-1*}$
Goodness-of-fit	1.08
Largest diff. peak and hole	0.72 and -0.34 e Å^{-3}

$^*P = (F_o^2 + 2F_c^2)/3$

Supplementary Table 2. Crystal data and structure refinement conditions for NiNb_propylene

Empirical formula	$C_{10.54}H_{13.07}F_5N_4NbNiO$
Formula weight	458.42
Crystal system, space group	Tetragonal, *P*4/*nbm*
Unit cell dimensions	a = 9.9888(3) Å, c = 7.9029(3) Å
Volume	788.52(6) $Å^3$
Z, calculated density	2, 1.931 Mg m^{-3}
F(000)	453
Temperature (K)	296(2)
Radiation type, λ	Cu $K\alpha$, 1.54178 Å
Absorption coefficient	7.99 mm^{-1}
Absorption correction	Multi-scan
Max and min transmission	0.148 and 0.037
Crystal size	0.005 × 0.02 × 0.02 mm
Shape, colour	Prism, colourless
θ range for data collection	6.3–65.9°
Limiting indices	$-11 \leq h \leq 11$, $-8 \leq k \leq 11$, $-9 \leq l \leq 8$
Reflection collected / unique / observed with $I > 2\sigma(I)$	3823 / 380 (R_{int} = 0.042) / 314
Completeness to θ_{max} = 65.9°	99.0 %
Refinement method	Full-matrix least-squares on F^2
Data / restraints / parameters	380 / 37 / 66
Final R indices [$I > 2\sigma(I)$]	R_1 = 0.030, wR_2 = 0.077
Final R indices (all data)	R_1 = 0.034, wR_2 = 0.079
Weighting scheme	$[\sigma^2(F_o^2) + (0.0425P)^2]^{-1*}$
Goodness-of-fit	1.15
Largest diff. peak and hole	0.48 and -0.21 e $Å^{-3}$

$^*P = (F_o^2 + 2F_c^2)/3$

Supplementary Table 3. Crystal data and structure refinement conditions for NiNb_propane

Empirical formula	$C_{10.42}H_{14.46}F_5N_4NbNiO$
Formula weight	458.38
Crystal system, space group	Tetragonal, *P*4/*mmm*
Unit cell dimensions	a = 7.0912(3) Å, c = 7.9658(6) Å
Volume	400.56(5) $Å^3$
Z, calculated density	1, 1.900 Mg m^{-3}
F(000)	227
Temperature (K)	296(2)
Radiation type, λ	Cu $K\alpha$, 1.54178 Å
Absorption coefficient	7.86 mm^{-1}
Absorption correction	Multi-scan
Max and min transmission	0.148 and 0.039
Crystal size	0.005 × 0.02 × 0.02 mm
Shape, colour	Prism, colourless
θ range for data collection	5.5–66.1°
Limiting indices	$-8 \leq h \leq 8$, $-8 \leq k \leq 8$, $-9 \leq l \leq 8$
Reflection collected / unique / observed with $I > 2\sigma(I)$	3798 / 245 (R_{int} = 0.052) / 224
Completeness to θ_{max} = 66.1°	98.8 %
Refinement method	Full-matrix least-squares on F^2
Data / restraints / parameters	245 / 21 / 46
Final R indices [$I > 2\sigma(I)$]	R_1 = 0.030, wR_2 = 0.071
Final R indices (all data)	R_1 = 0.033, wR_2 = 0.072
Weighting scheme	$[\sigma^2(F_o^2) + (0.0360P)^2 + 0.2536P]^{-1*}$
Goodness-of-fit	1.19
Largest diff. peak and hole	0.48 and -0.19 e $Å^{-3}$

$^*P = (F_o^2 + 2F_c^2)/3$

Supplementary Table 4. Parameters of C–H···F contacts in experimental structures

NiNb_empty

Contact	*d*(C–H), Å	*d*(H···F), Å	*d*(C···F), Å	∠(C–H···F), °
		Intramolecular		
C1–H1···F2	0.93	2.53	3.45(2)	169.9
C1–H1···F2^{i}	0.93	2.41	3.30(2)	159.3

Symmetry code: (i) = ½-*x*, *y*, -z.

NiNb_propylene

Contact	*d*(C–H), Å	*d*(H···F), Å	*d*(C···F), Å	∠(C–H···F), °
		Intramolecular		
C1A–H1A···F2A	0.93	2.40	3.30(1)	162.9
C1A–H1A···F2B^{i}	0.93	2.47	3.14(1)	128.8
C1A–H1A···F2A^{ii}	0.93	2.61	3.54(1)	176.7
C1B–H1B···F2B	0.93	2.40	3.18(3)	140.7
		Intermolecular		
C2P–H2P···F2B^{ii}	0.93	2.47	3.13(3)	128.4
C3P–H3PA···F2A^{iii}	0.96	2.49	3.167(9)	127.7
C3P–H3PA···F2A^{iv}	0.96	2.65	3.153(8)	112.8
C3P–H3PC···F2A	0.96	2.51	3.153(8)	124.0
C3P–H3PC···F2A^{ii}	0.96	2.65	3.167(9)	114.2

Symmetry code: (i) = *y*, *x*, 1-*z*; (ii) = *x*, ½-*y*, 1-*z*; (iii) = ½+*y*, 1-*x*, 1-*z*; (iv) = 1-*y*, 1-*x*, *z*.

NiNb_propane

Contact	*d*(C–H), Å	*d*(H···F), Å	*d*(C···F), Å	∠(C–H···F), °
		Intramolecular		
C1–H1···F2A^{i}	0.93	2.39	3.150(7)	138.9
C1–H1···F2B	0.93	2.36	3.28(3)	170.8
		Intermolecular		
C1P–H1PB···F2B^{ii}	0.96	2.52	3.01(7)	111.2
C2P–H2PA···F2A	0.97	2.45	3.19(5)	133.6
C2P–H2PA···F2B^{iii}	0.97	2.47	3.15(1)	127.3

Symmetry code: (i) = *x*, 1-*y*, 1-z; (ii) = -*x*, -*y*, *z*; (iii) = -*x*, *y*, 1-z.

Supplementary Table 5. Comparison of the calculated and experimental empty host structures. The unit cell parameters (see Supplementary Scheme 2), the absolute energies, anion orientations φ and pyrazine tilt angles θ for calculated structures with different ordering of neighbouring Ni-pyrazine layers. The analogous figures are also shown for experimental structures of **NbOFFIVE**-1-Ni and **SIFSIX**-3-Ni for comparison. The lowest energy configurations for each material are highlighted in grey. The reported experimental values for **NbOFFIVE**-1-Ni are from single crystal data reported in this work, while the figures for **SIFSIX**-3-Ni are from Rietveld refinement of PXRD data reported in [Elsaidi, S. K. *et al.*, *Chem Sci* **8**, 2373, (2017)].

	a, Å	*c*, Å	*V*, $Å^3$	*E*, eV	φ,˚	θ,˚
**NbOFFIVE**-1-Ni opposite	9.853	15.649	1519.26	-605.016	9.8	29.2
**NbOFFIVE**-1-Ni identical	9.843	15.611	1512.41	-605.109	10.2	29.2
**NbOFFIVE**-1-Ni experiment	9.929	15.662	1544.08	N/A	7.5	27.5
**SIFSIX**-3-Ni opposite	9.791	14.906	1428.81	-579.811	0	25.9
**SIFSIX**-3-Ni identical	9.798	14.986	1438.65	-579.756	0	25.5
**SIFSIX**-3-Ni experiment	9.901	14.996	1470.11	N/A	0	18.3

Supplementary Table 6. The properties of the calculated adsorption sites for propylene and propane in NbOFFIVE-1-Ni and SIFSIX-3-Ni. The unit cell volume, the absolute energies, adsorption energies E_{ads} and the three contributions to the adsorption energies calculated for the configurations discussed in the main text. The absolute energies of propylene and propane in the gas phase are respectively -44.333 eV and -52.492 eV. The lowest energy configurations for each material/guest combination (the global minimum) are highlighted in grey.

	V, $Å^3$	*E*, eV	E_{ads}, kJ/mol	Strain host	Strain guest	host-guest attraction
**NbOFFIVE**-1-Ni propylene, **A**	1526.87	-650.040	-57.7	8.7	0.9	-67.3
**NbOFFIVE**-1-Ni propylene, **B′**	1517.53	-650.218	-74.8	4.8	0.6	-80.3
**NbOFFIVE**-1-Ni propane, **C**	1530.29	-658.128	-50.8	18.0	1.8	-70.7
**NbOFFIVE**-1-Ni propane, **D′**	1534.74	-658.090	-47.2	19.6	0.5	-67.3
**NbOFFIVE**-1-Ni propane, **E**	1521.65	-658.215	-59.2	21.9	2.5	-83.6
**SIFSIX**-3-Ni propylene, **A**	1438.97	-624.822	-65.5	6.9	0.8	-73.2
**SIFSIX**-3-Ni propylene, **B**	1435.70	-624.952	-78.0	3.6	1.3	-82.9
**SIFSIX**-3-Ni propane, **C** and **D**	1443.95	-632.981	-65.5	11.9	0.7	-78.1
**SIFSIX**-3-Ni propane, **E**	1444.91	-632.948	-62.3	10.1	1.3	-73.6

Supplementary Table 7. The effect of the framework dynamics on the calculated adsorption energy. Time-averaged adsorption energies calculated at 300K in *ab initio* MD calculations, $E_{\text{ads at 300K}}$, in a fixed unit cell with the dimensions of that observed experimentally for the desolvated material and given in Supplementary Table 5. The adsorption energies of the global minimum configuration, E_{ads}, listed in Supplementary Table 6 and the energy difference, $\Delta E = E_{\text{ads at 300K}} - E_{\text{ads}}$, due to thermal motion are also shown for comparison.

	$E_{\text{ads at 300K}}$, kJ/mol	E_{ads}, kJ/mol	ΔE, kJ/mol
**NbOFFIVE**-1-Ni propylene	-58.0 ± 1.8	-74.8	16.8
**NbOFFIVE**-1-Ni propane	-45.7 ± 1.9	-59.2	13.5
**SIFSIX**-3-Ni propylene	-66.7 ± 1.7	-78.0	11.3
**SIFSIX**-3-Ni propane	-58.6 ± 1.9	-65.5	6.9

Supplementary References

[1] G. Kresse and J. Hafner, *Phys. Rev. B*, **47**, 558, (1993).
[2] Klimeš, D. R. Bowler, and A. Michaelides, J. Phys.: Cond. Matt. **22**, 022201 (2010).
[3] P. E. Blochl. Projector augmented-wave method. *Phys. Rev. B*, **50**, 17953, (1994).
[4] J. P. Perdew, K. Burke, and M. Ernzerhof, *Phys. Rev. Lett.*, **77**, 3865, (1996).
